# Associations between outdoor light exposure and low muscle mass: An NHANES 2011 to 2014 analysis

**DOI:** 10.1097/MD.0000000000048257

**Published:** 2026-04-10

**Authors:** Zhiwei Xue, Dong Sun, Zhaolin Wang, Dong Yan, Feifei Deng, Zhenyuan Yu, Peng Liu

**Affiliations:** a Department of Orthopaedics, China-Japan Union Hospital of Jilin University, Changchun, Jilin, China.

**Keywords:** ActiGraph GT3X+ accelerometer, outdoor light exposure, sarcopenia, testosterone, vitamin D

## Abstract

Despite the focus of most studies on the association between ultraviolet B radiation or vitamin D and sarcopenia, the controversy still exists. The aim of this study was to explore the association between time spent outdoors (TSO) and outdoor light intensity (OLI) and the odds of sarcopenia. This study employed the National Health and Nutrition Examination Survey data in 2011 to 2014 period that included ActiGraph GT3X+ accelerometer data. Multiple logistic regression and restricted cubic spline curve were used to explore the possible association between TSO and OLI with sarcopenia. Mixed mediation analysis was used to explore possible mechanisms. Subgroup analysis and sensitivity analysis were conducted. After adjusting for all covariates, TSO and OLI were negatively associated with sarcopenia (TSO: odds ratio = 0.80 [0.68–0.94]; OLI: odds ratio = 0.78 [0.66–0.94]). A considerable part of these relationships was explained by vitamin D (9.49% for TSO; 8.44% for OLI) and testosterone (6.82% for TSO; 5.14% for OLI). The chain mediation path of “vitamin D → testosterone” mediated 1% of the association between the 2 variables, TSO and OLI, with sarcopenia. TSO and OLI were negatively associated with sarcopenia. Vitamin D and testosterone, parallel and chain, mediated this negative association.

## 1. Introduction

Sarcopenia is a progressive skeletal muscle disorder with rapid loss of skeletal muscle mass.^[1,2]^ It is frequently accompanied by chronic comorbidities, such as diabetes, arthritis, and cancer.^[3–5]^ Sarcopenia is highly prevalent in the elderly population. The prevalence of sarcopenia in the elderly aged 60 to 70 was 5% to 13%, and increased to 50% in the elderly over 80 years of age.^[6]^ The prevalence of sarcopenia in the general population worldwide has been reported to be 5% to 10%.^[7]^ Sarcopenia impairs physical function and activity capacity, significantly increasing the risk of adverse events.^[1,2]^ Adverse events include falls, fractures, muscle weakness, quality of life, and mortality.^[1,3,7]^ Therefore, early detection of sarcopenia is important for effective treatment and preventing disease progression.^[8]^

For decades, sun exposure has been blamed by the media and dermatological associations due to its association with increased cancer risk.^[9]^ In addition, sun exposure confers many benefits on human health via vitamin D synthesis, regulation of circadian rhythms, and other pathways.^[9]^ In recent years, increased exposure to the sun has been associated with a reduced risk of low testosterone, and higher testosterone levels are associated with better quality of life and well-being.^[10]^ Most of the aforementioned studies focused on the association between artificial ultraviolet B (UVB) radiation or vitamin D and sarcopenia, and controversy exists regarding the association.^[11–13]^ In a Korean cohort study, higher 25-hydroxyvitamin D (25[OH]D) levels were associated with a lower likelihood of muscle mass loss.^[14]^ In contrast, an animal model study demonstrated that the beneficial effect of ultraviolet exposure on sarcopenia is dependent on vitamin D deficiency.^[15]^ No significant differences in muscle strength or mass were observed among animals with or without ultraviolet exposure in vitamin D-sufficient groups.^[15]^ However, studies focusing on the association between time spent outdoors (TSO) and outdoor light intensity (OLI) and low muscle mass (LMM) are scarce. We hypothesized that greater TSO and higher OLI would be associated with lower odds of LMM, and that this association would be partially mediated by vitamin D and testosterone.

## 2. Methods

### 2.1. Study participants

**Figure 1.**
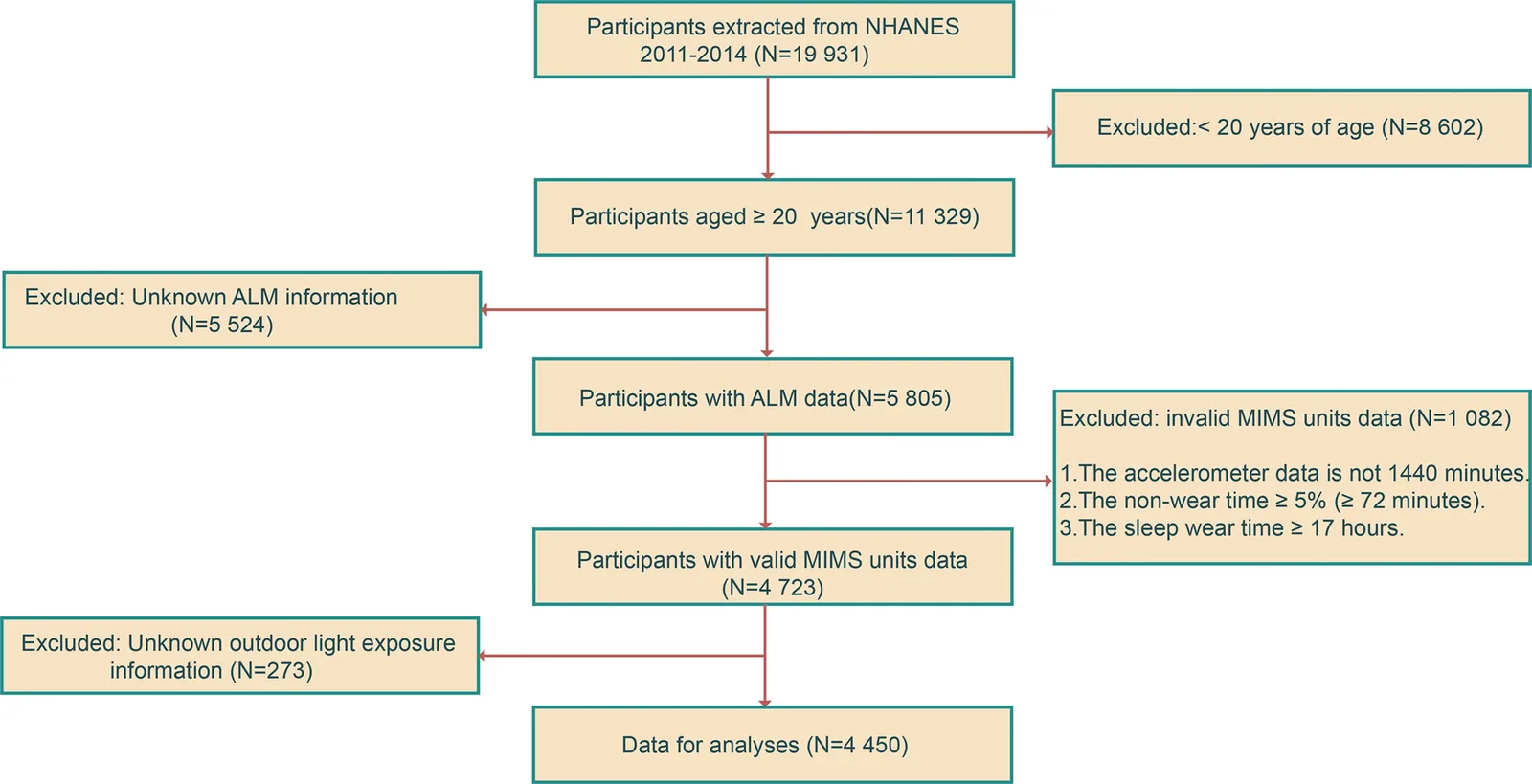
Flowchart of participants selection. ALM = appendicular lean mass, MIMS = monitor independent movement summary, NHANES = National Health and Nutrition Examination Survey.

This research used data from the National Health and Nutrition Examination Survey (NHANES) database. NHANES is designed to examine the nutritional and health status of Americans. ActiGraph GT3X+ wrist-worn accelerometers data are only available in the 2011 to 2014 period of the NHANES database. Eligible participants were adults aged ≥20 years from the NHANES 2011 to 2014 cycles with valid accelerometer data, appendicular lean mass measurements, and outdoor light exposure information. The original sample was 19,931 subjects. After exclusion, our final analysis sample comprised 4450 subjects (Fig. 1).

### 2.2. Physical activity metrics

During the 2011 to 2014 NHANES cycle, researchers used the wrist-worn ActiGraph GT3X+ accelerometer to monitor participants’ physical activity patterns for 7 consecutive days, 24 hours per day.^[16]^ Participants were instructed to wear the accelerometer on their nondominant wrist to record raw triaxial (X, Y, Z) acceleration data aggregated at 80 Hz.^[17,18]^ NHANES utilized monitor independent movement summary (MIMS) units derived from processing the raw data through algorithms.^[17,18]^ Data from the first and last days were not usable.^[19]^ A valid wear day was defined as having a wear time of 1440 minutes, with nonwear time accounting for <5%, and a recorded sleep time of <17 hours.^[18]^ Participants with at least 1 valid day of data were included in the analysis.^[20]^ Daily MIMS (MIMS/d × 10^4^) was calculated as the mean of the sum of minute-level MIMS units across all valid days.^[21]^ The ActiGraph GT3X+ accelerometer also collected ambient light data at a sampling frequency of 1 Hz.^[16,19]^ These second-level lux values were summarized into minute-level averages and stored in the “PAXLUX” variable. Studies have defined a minute with an average lux value >240 as an “outdoor minute.”^[19]^ However, the period between 10:00 pm and 6:00 am was excluded because typical outdoor light levels during the night would be misclassified by the 240 lux threshold.^[22]^ TSO (TSO/h) was calculated by averaging the total number of “outdoor minutes” across each participant’s valid days. As some studies have used a threshold of >500 average lux per minute to define an “outdoor minute,” we used the 500 lux threshold for validation in a sensitivity analysis.^[19]^

### 2.3. Low muscle mass

Dual-energy X-ray absorptiometry is the gold standard for measuring appendicular skeletal muscle mass (ASM).^[23]^ Although magnetic resonance imaging is the gold standard for assessing ASM, appendicular lean mass measured by dual-energy X-ray absorptiometry serves as a cost-effective and practical alternative.^[24]^ In this study, the diagnostic criteria for LMM were based on the guidelines established by the Foundation for the National Institutes of Health in 2014.^[23,24]^ According to the Foundation for the National Institutes of Health criteria, participants were classified as having LMM if their ASM/body mass index ratio was <0.789 for men and <0.512 for women.^[25]^

### 2.4. Vitamin D

The outcome variable in this study was serum total 25(OH)D, which represents the sum of the concentrations of 25(OH)D2 and 25(OH)D3.^[26]^ Ultra-performance liquid chromatography-tandem mass spectrometry was used to measure serum total vitamin D levels.^[27,28]^ According to the Endocrine Society Clinical Practice Guidelines, vitamin D status was categorized into 3 groups: deficiency (<50.0 nmol/L), insufficiency (50.0–74.9 nmol/L), and sufficiency (≥75.0 nmol/L).^[29]^

### 2.5. Testosterone

Testosterone was included as a mediator for both sexes because it plays a role in muscle maintenance in men and women, albeit at different physiological levels.^[30,31]^ Participants fasted overnight prior to serum testosterone sampling, and all other blood samples were drawn between 8:30 and 11:30 am to reduce metabolic effects and for reasons of diurnal variation.^[32]^ Serum total testosterone was measured by highly accurate isotope dilution liquid chromatography-tandem mass spectrometry.^[32,33]^ Free testosterone was calculated using the Vermeulen formula.^[34,35]^

### 2.6. Covariates

The covariates we selected were sex, age, race, educational level, marital status, Daily MIMS, cancer, arthritis, history of diabetes, and smoking. Full details about the calculation, measurement, and interpretation of these variables are available on the official NHANES website. Daily MIMS represents the average daily activity level.

### 2.7. Statistical analysis

Because a complex survey sampling design was used, weighted analyses according to NHANES recommended algorithms (WTMEC2YR) were conducted. Continuous variables were presented as means (standard deviations), and categorical variables as counts (percentages). Before formal analysis, we explored the bivariate correlations among all continuous variables using Pearson correlation. We used multivariable weighted logistic regression models to investigate the association between outdoor light exposure and sarcopenia. We added covariates into models step by step as follows: model I was not adjusted; model II was adjusted for basic demographic characteristics, including age, sex, race, educational level, and marital status; and model III was further adjusted for history of diabetes, smoking, arthritis, and cancer. We used restricted cubic spline (RCS) models with 3 knots to test the nonlinear relationship between outdoor light exposure and sarcopenia. We performed subgroup analyses and explored the possible interaction using a likelihood ratio test. Mediation analysis was conducted under the assumptions that outdoor light exposure is associated with the mediators (vitamin D and testosterone), the mediators are associated with LMM after controlling for outdoor light exposure, and outdoor light exposure affects LMM indirectly through the mediators. We used mixed mediation models (including chain mediation and parallel mediation models) to explore the possible underlying mechanism. Finally, we performed a sensitivity analysis by changing the outdoor light exposure threshold to examine the robustness of the study’s primary findings.

## 3. Results

### 3.1. Baseline characteristics

This study ultimately included 4450 participants. Based on the weighted analysis, 329 individuals (7.4%) were identified as having LMM. As shown in Table 1, compared with the normal muscle mass group, participants in the LMM group were older. The prevalence of diabetes history and arthritis was also significantly higher in the LMM group. Furthermore, the LMM group exhibited significantly lower levels of vitamin D, physical activity (Daily MIMS), as well as TSO and OLI based on 2 different light thresholds (*P* ≤ .001).

**Table 1:** Survey-weighted baseline characteristics of the study population.

	Overall (N = 4450)	Normal muscle mass (n = 4121)	Low muscle mass (n = 329)	*P* value
Age (yr)	40.19 ± 11.63	39.82 ± 11.57	44.99 ± 11.35	<.001
Gender (%)				.028
Male	2208 (50%)	2045 (50%)	163 (57%)	
Female	2242 (50%)	2076 (50%)	166 (43%)	
Race (%)				<.001
Mexican American	558 (9.5%)	468 (8.7%)	90 (20%)	
Other Hispanic	401 (6.5%)	352 (6.2%)	49 (10%)	
Non-Hispanic White	1729 (64%)	1617 (64%)	112 (57%)	
Non-Hispanic Black	1002 (12%)	975 (12%)	27 (4.2%)	
Other Races	760 (8.5%)	709 (8.6%)	51 (7.8%)	
Educational level (%)				.001
Less than high school	800 (14%)	713 (13%)	87 (22%)	
High school or GED	951 (21%)	866 (20%)	85 (26%)	
Above high school	2699 (65%)	2542 (66%)	157 (52%)	
Marital status (%)				.379
Married or cohabiting	2601 (62%)	2402 (63%)	199 (59%)	
Other	1849 (38%)	1719 (37%)	130 (41%)	
History of diabetes (%)				<.001
No	4118 (94%)	3841 (95%)	277 (87%)	
Yes	332 (5.9%)	280 (5.3%)	52 (13%)	
Smoking (%)				.635
No	2648 (58%)	2448 (58%)	200 (56%)	
Yes	1802 (42%)	1673 (42%)	129 (44%)	
Arthritis (%)				<.001
No	3801 (84%)	3544 (85%)	257 (76%)	
Yes	649 (16%)	577 (15%)	72 (24%)	
Cancer (%)				.275
No	4265 (94%)	3955 (94%)	310 (92%)	
Yes	185 (5.8%)	166 (5.6%)	19 (7.7%)	
Vitamin D (nmol/L)	66.11 ± 25.87	66.71 ± 25.95	58.23 ± 23.51	<.001
Testosterone (ng/dL)	219.71 ± 228.36	222.32 ± 231.55	184.86 ± 177.22	.009
Daily MIMS (MIMS/d × 10^4^)	1.42 ± 0.36	1.42 ± 0.36	1.32 ± 0.41	<.001
Time spent outdoors (h), threshold > 240 lux	2.14 ± 1.48	2.17 ± 1.48	1.80 ± 1.36	.001
Time spent outdoors (h), threshold > 500 lux	1.66 ± 1.28	1.69 ± 1.29	1.36 ± 1.05	.001
Outdoor light intensity (lux), threshold > 240 lux	1.62 ± 1.43	1.65 ± 1.44	1.29 ± 1.20	.001
Outdoor light intensity (lux), threshold > 500 lux	1.51 ± 1.39	1.53 ± 1.40	1.19 ± 1.17	.001

GED = general educational development, MIMS = monitor independent movement summary.

### 3.2. Survey-weighted association of TSO and OLI with the odds of LMM

As shown in Table 2, in the 3 models, TSO and OLI demonstrated significant negative associations with the odds of LMM. In the fully adjusted model (model II), greater TSO was significantly associated with reduced odds of LMM (odds ratio [OR] = 0.80 [0.68–0.94]). Participants in the highest quartile (Q4) exhibited a significantly 63% lower odds of LMM compared with those in the lowest quartile (Q1) (OR = 0.37 [0.21–0.65]). Higher OLI was significantly associated with lower odds of LMM (OR = 0.78 [0.66–0.94]). Compared with the Q1 group, the Q4 group had significantly reduced odds of LMM (OR = 0.35 [0.20–0.60]).

**Table 2:** The association of weighted TSO and OLI with the odds of LMM.

	Crude model*		Model I†		Model II‡	
	OR (95% CI)	*P* value	OR (95% CI)	*P* value	OR (95% CI)	*P* value
TSO	0.82 (0.71–0.94)	.006	0.76 (0.65–0.88)	<.001	0.80 (0.68–0.94)	.008
Q1	Ref	–	Ref	–	Ref	–
Q2	0.66 (0.46–0.94)	.025	0.67 (0.45–1.01)	.054	0.71 (0.46–1.09)	.110
Q3	0.69 (0.47–1.01)	.058	0.61 (0.38–0.97)	.037	0.68 (0.42–1.12)	.120
Q4	0.40 (0.25–0.64)	<.001	0.30 (0.18–0.52)	<.001	0.37 (0.21–0.65)	.002
OLI	0.80 (0.68–0.94)	.009	0.74 (0.62–0.89)	.002	0.78 (0.66–0.94)	.011
Q1	Ref	–	Ref	–	Ref	–
Q2	0.54 (0.35–0.84)	.008	0.51 (0.33–0.80)	.005	0.54 (0.33–0.87)	.014
Q3	0.75 (0.52, 1.08)	.120	0.65 (0.41–1.01)	.056	0.73 (0.46–1.15)	.200
Q4	0.38 (0.24–0.60)	<.001	0.29 (0.17–0.48)	<.001	0.35 (0.20–0.60)	<.001

CI = confidence interval, LMM = low muscle mass, MIMS = monitor independent movement summary, OLI = outdoor light intensity, OR = odds ratio, Q = quartile, TSO = time spent outdoors.

* Crude model: no covariates were adjusted.

† Model I: sex, age, race, educational level, and marital status were adjusted.

‡ Model II: sex, age, race, educational level, marital status, cancer, arthritis, Daily MIMS, history of diabetes, and smoking were adjusted.

### 3.3. Nonlinearity analysis using RCS

**Figure 2.**
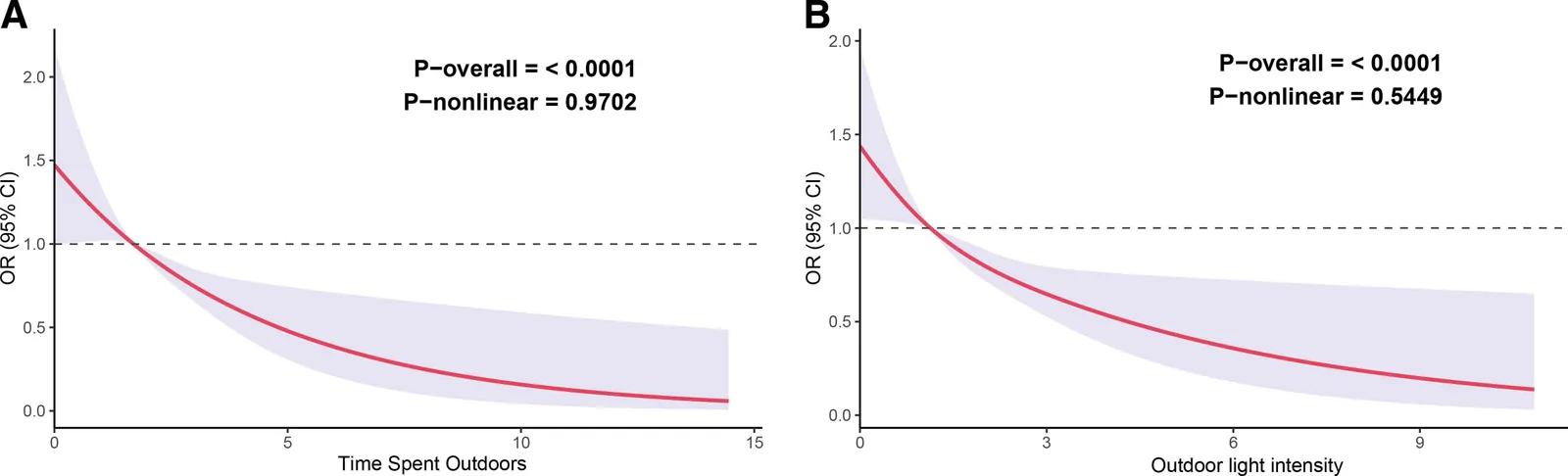
The RCS curve of TSO (A), OLI (B) with the odds of low muscle mass. RCS regression was fully adjusted. CI = confidence interval, OLI = outdoor light intensity, OR = odds ratio, RCS = restricted cubic spline, TSO = time spent outdoors.

RCS analysis revealed nonlinear associations between TSO and OLI with the odds of LMM. As shown in Figure 2, significant associations were observed between both TSO and OLI with the odds of LMM. However, no significant nonlinear trend was detected. Higher levels of TSO and OLI exhibit a linear dose-response relationship with lower odds of LMM.

### 3.4. Subgroup analysis

**Figure 3.**
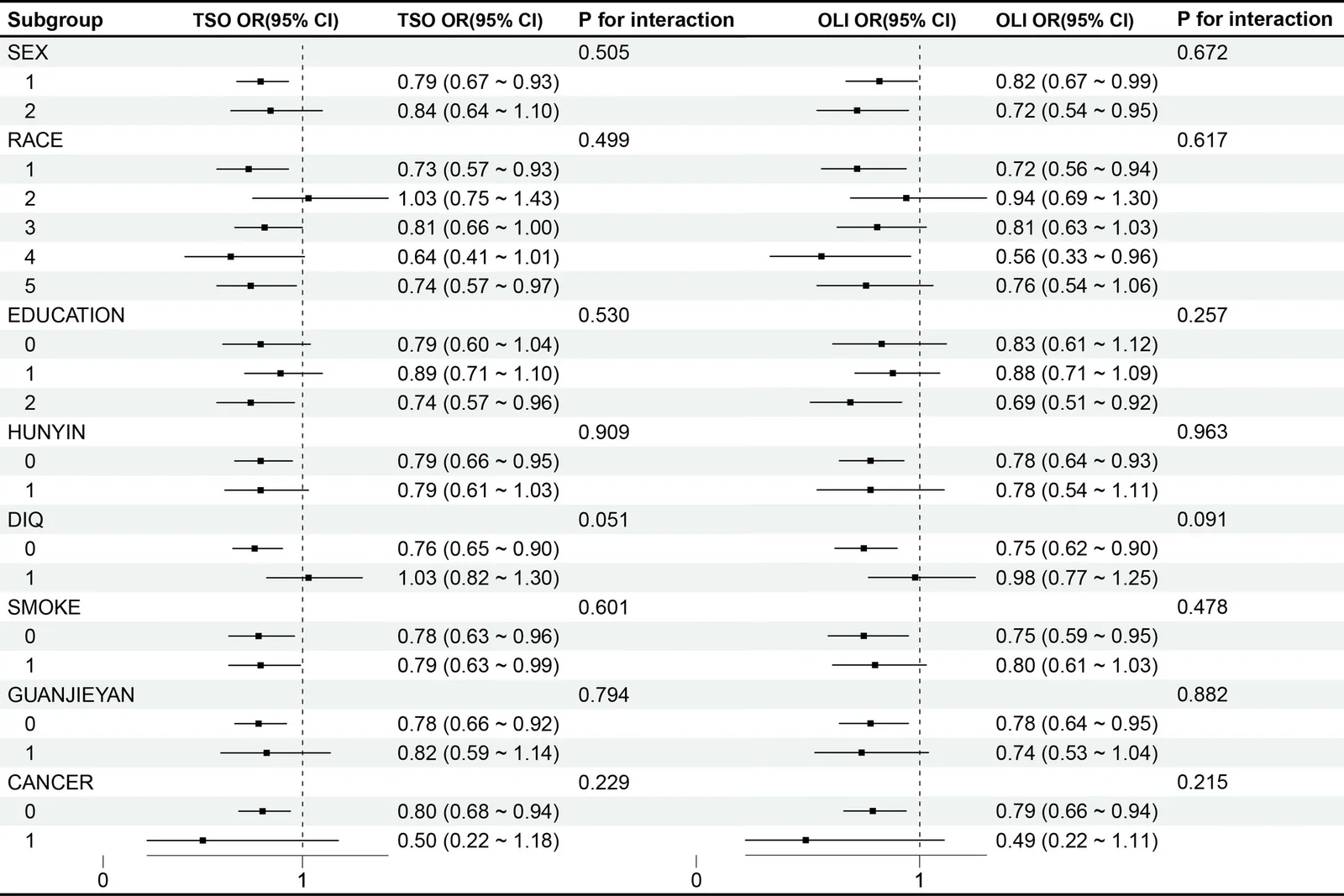
Subgroup analysis for the association between TSO and OLI with the odds of LMM. CI = confidence interval, LMM = low muscle mass, OLI = outdoor light intensity, OR = odds ratio, TSO = time spent outdoors.

Subgroup analyses were conducted to assess the consistency of the associations between TSO and OLI with the odds of LMM across different groups (Fig. 3). Negative associations between TSO and OLI with LMM remained consistent across all subgroups in this study (*P* for interaction > .05).

### 3.5. Mediation analysis

**Figure 4.**
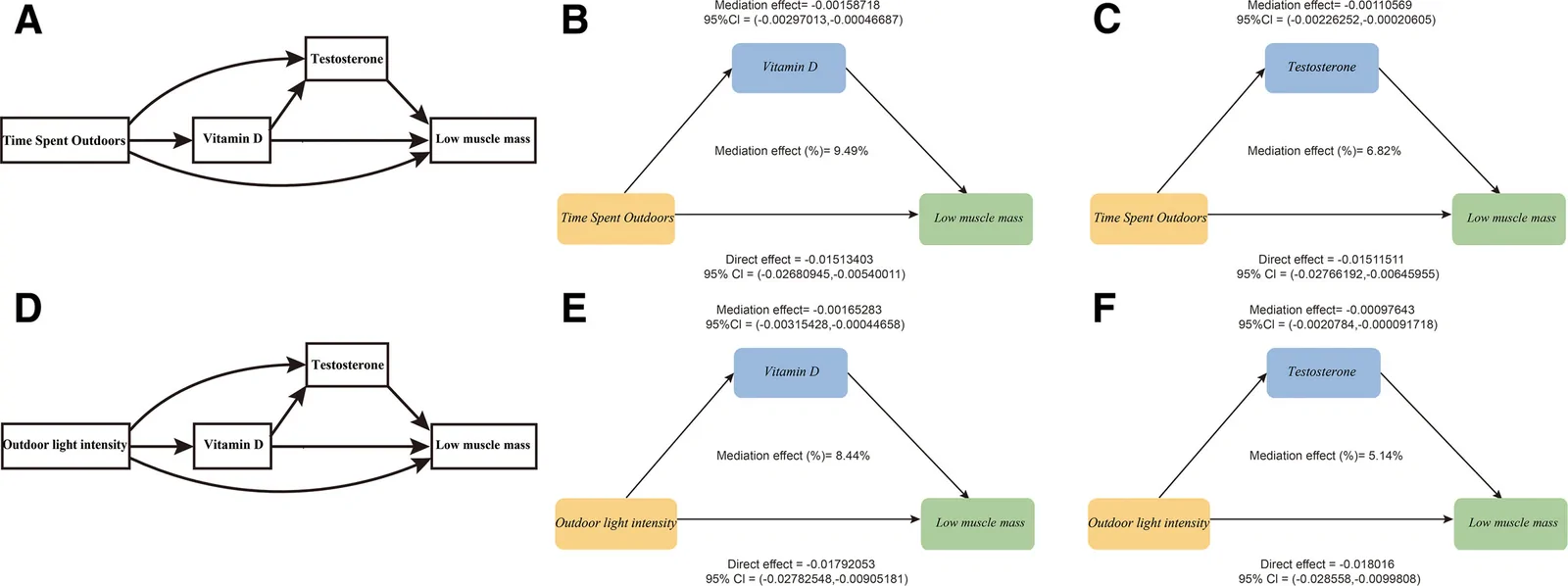
The results of the mediation analysis. (A) Path diagram of the mixed mediation analysis models of TSO and LMM. (B) Vitamin D. (C) Testosterone partially mediates the relationship between TSO and LMM. (D) Path diagram of the mixed mediation analysis models of OLI and LMM. (E) Vitamin D. (F) Testosterone partially mediates the relationship between OLI and LMM. CI = confidence interval, LMM = low muscle mass, OLI = outdoor light intensity, OR = odds ratio, TSO = time spent outdoors.

Bivariate correlations indicated that both TSO and OLI were significantly correlated with vitamin D (TSO: *r* = 0.17, *P* < .001; OLI: *r* = 0.18, *P* < .001) and testosterone (TSO: *r* = 0.22, *P* < .001; OLI: *r* = 0.22, *P* < .001), providing preliminary support for their roles as potential mediators (Fig. S1, Supplemental Digital Content, https://links.lww.com/MD/R637). The mixed mediation models were constructed. TSO and OLI served as independent variables, while vitamin D and testosterone acted as mediators, with LMM as the dependent variable. The result shows that both vitamin D and testosterone mediate the association between TSO and LMM (*P* < .05) (Fig. 4). Vitamin D and testosterone explained 9.49% and 6.82% of the association between TSO and LMM, respectively (vitamin D: indirect effect [IE] = −0.00158718 [−0.00297013, −0.00046687]; testosterone: IE = −0.00110569 [−0.00226252, −0.00020605]). Vitamin D and testosterone explained 8.44% and 5.14% of the association between OLI and LMM, respectively (vitamin D: IE = −0.00165283 [−0.00315428, −0.00044658]; testosterone: IE = −0.00097643 [−0.0020784, −0.000091718]). The chain mediation path of “vitamin D → testosterone” mediated 1% of the association between both TSO and OLI with LMM (*P* < .05). More detailed information is in Table S1, Supplemental Digital Content, https://links.lww.com/MD/R638.

### 3.6. Sensitivity analyses

For sensitivity analysis, we defined an “outdoor minute” as >500 lux/min (average). After fully adjusting for covariates, it was found that the recalculated TSO and OLI still showed significant negative associations with LMM (Table S2, Supplemental Digital Content, https://links.lww.com/MD/R638). When dividing TSO and OLI into 4 equal parts, the relationship was also validated. The results have a certain degree of robustness. The mixed mediation results remain significant (Fig. S2, Supplemental Digital Content, https://links.lww.com/MD/R637).

## 4. Discussion

This study investigated the associations between TSO and OLI with LMM. The results indicated that both higher levels of TSO and OLI were positively associated with the odds of LMM. These findings remained robust across subgroup and sensitivity analyses. Importantly, 3 models were rigorously adjusted for various confounding factors, including device-measured overall physical activity levels. This suggested that increased outdoor light exposure may confer a direct protective effect on muscle health. These findings suggest that increasing outdoor light exposure may contribute to muscle preservation, offering a potential nonpharmacological target for sarcopenia prevention. Further interventional studies are needed to confirm causality.

Bhambhvani et al first demonstrated that longer TSO is associated with higher serum testosterone levels and lower odds of testosterone deficiency.^[10]^ Testosterone is crucial for maintaining muscle mass.^[36]^ However, research directly examining the association between outdoor light exposure and muscle mass remains scarce. This study is the first to investigate this association, providing evidence for the association between environmental exposure and muscle health outcomes. Promoting greater outdoor light exposure among the general public appears to be a clinically reasonable recommendation with proven benefits for muscle.

Sarcopenia is associated with a multitude of adverse health consequences.^[37]^ Given its pathophysiology, sarcopenia is characterized by the net imbalance between muscle anabolism and catabolism and possibly neuronal degeneration.^[3]^ That is, sarcopenia is caused by impairment of the neuromuscular junction, dysregulation of calcium metabolism, a decrease in the number of muscle satellite cells, infiltration of inflammatory cells, and mitochondrial dysfunction causing metabolic abnormalities such as insulin resistance, which leads to excess muscle protein degradation over synthesis and aggravation of muscle wasting.^[38–40]^ Sufficient dietary vitamin D intake is associated with a positive effect on sarcopenia.^[41]^ However, similar to dietary intake, other environmental exposures – particularly outdoor light – are emerging with unique characteristics potentially conferring novel significance. Solar UVB radiation is the main source of vitamin D in humans, which accounts for only a minor proportion of dietary intake.^[42,43]^ In recent years, a “skin-brain-gonad” axis activated by sun exposure has been elucidated: irradiation of the skin upon UVB exposure sends signals to the brain and then increases secretion of testosterone and estrogen from the gonads through a p53 protein-dependent process in the brain; testosterone and estrogen produced by the gonads play important roles in the maintenance and promotion of muscle mass.^[44,45]^ It was reported that exposure for about 25 minutes at solar noon induced significantly positive activation of upstream regulatory genes related to testosterone in both men and women.^[46]^ Testosterone increases muscle mass by promoting muscle protein synthesis.^[36]^

The study found that vitamin D mediated the association between outdoor light exposure and odds of LMM. The effects of vitamin D on muscle have been extensively studied. Vitamin D can stimulate the proliferation and differentiation of skeletal muscle fibers to maintain and improve muscle strength and physical performance.^[47]^ It aligns with our findings. Vitamin D receptors are expressed in various muscle cell models, including C2C12 and L6 cells. Its active form, 1,25-dihydroxyvitamin D3, participates in regulating myogenesis, cell proliferation, differentiation, protein synthesis, and mitochondrial metabolism by activating signaling cascades, including the mitogen-activated protein kinase pathway.^[48]^ Experimental evidence shows that 1,25-dihydroxyvitamin D3 exerts protective effects against drug-induced mitochondrial toxicity in myotubes,^[49]^ and a 3-month diet-induced vitamin D deficiency reduced mitochondrial respiratory function in mouse skeletal muscle, which may underlie the muscle fatigue associated with vitamin D deficiency.^[50]^

The mediation analysis also revealed that testosterone mediated the association between outdoor light exposure and the odds of LMM. Greater TSO is associated with higher serum testosterone levels.^[10]^ The anabolic effects of testosterone on muscle are well established.^[51]^ It directly increases muscle mass by enhancing muscle protein synthesis.^[36]^ A meta-analysisincorporating 13 observational studies conclusively indicated that decreased endogenous testosterone levels increase the risk of sarcopenia in men.^[52]^ The underlying mechanisms may include altering the size of type I and II muscle fibers^[53]^; promoting protein synthesis by improving the efficiency of intracellular amino acid recycling^[54]^; stimulating satellite cell activity^[55]^; activating G protein-coupled receptors to increase intracellular calcium concentration^[56]^; and influencing muscle hypertrophy via increased insulin-like growth factor-1 expression.^[57]^

However, vitamin D and testosterone are not independent of each other. Supplementation with vitamin D increases testosterone levels, according to 1 study.^[58]^ The association of vitamin D with muscle strength is mediated by testosterone due to competition between the transcriptional targets of the androgen receptor and vitamin D receptor.^[13]^ This supports our finding of the chain mediation effect in this study. Vitamin D and testosterone play a sequential role in maintaining muscle health.

The strengths of this study include several aspects. First, the sample size was substantial and representative. Second, the use of objectively measured OLI (lux) via wrist-worn ActiGraph GT3X+ accelerometers is more precise than self-reported measures. Third, the concurrent inclusion of device-measured physical activity data allowed for a more effective isolation of the pure light exposure effect. Fourth, mixed mediation models were conducted to explore the underlying mechanisms, revealing a chain mediation effect from vitamin D to testosterone.

This study also has several limitations. First, the cross-sectional studies cannot clarify the possible causal relationship between TSO and OLI with the odds of LMM. Second, despite adjustment for numerous covariates, there are still some potential confounding factors that have not been included in the model, such as undiagnosed chronic diseases, seasonal variations, and geographic latitude. Finally, as NHANES only collected data over 7 consecutive days, the long-term dynamic effects could not be analyzed. Prospective cohort studies are warranted to further investigate these issues.

Future longitudinal and interventional studies are warranted to validate the causal relationship between outdoor light exposure and muscle mass. Research examining the optimal duration, timing, and intensity of outdoor light for muscle health across diverse populations is also recommended.

## 5. Conclusion

TSO and OLI were negatively associated with the odds of LMM. Vitamin D and testosterone, parallel and chain, mediate this negative association. It indicated that simply increasing outdoor light exposure itself may have a direct protective effect on muscle health, providing a novel and feasible nonpharmacological intervention target for the prevention and treatment of sarcopenia, which has important clinical and public health significance.

## Author contributions

**Conceptualization:** Zhiwei Xue.

**Data curation:** Zhiwei Xue, Dong Sun.

**Methodology:** Zhiwei Xue, Zhaolin Wang, Dong Yan.

**Software:** Zhiwei Xue, Feifei Deng.

**Formal analysis:** Dong Sun.

**Funding acquisition:** Dong Sun, Zhaolin Wang.

**Investigation:** Zhaolin Wang.

**Project administration:** Dong Yan.

**Resources:** Dong Yan, Feifei Deng.

**Supervision:** Feifei Deng.

**Validation:** Zhenyuan Yu.

**Visualization:** Zhenyuan Yu.

**Writing – review & editing:** Peng Liu.

**Writing – original draft:** Zhiwei Xue.

## Supplementary Material




