# Supplementary material for: Associations between outdoor light exposure and low muscle mass: An NHANES 2011 to 2014 analysis
Source: Medicine (Baltimore). 2026 Apr 10;105(15):e48257. doi: 10.1097/MD.0000000000048257 (PMC13593248; doi:10.1097/MD.0000000000048257)

Supplementary Figure 1. The result of pearson's correlation.

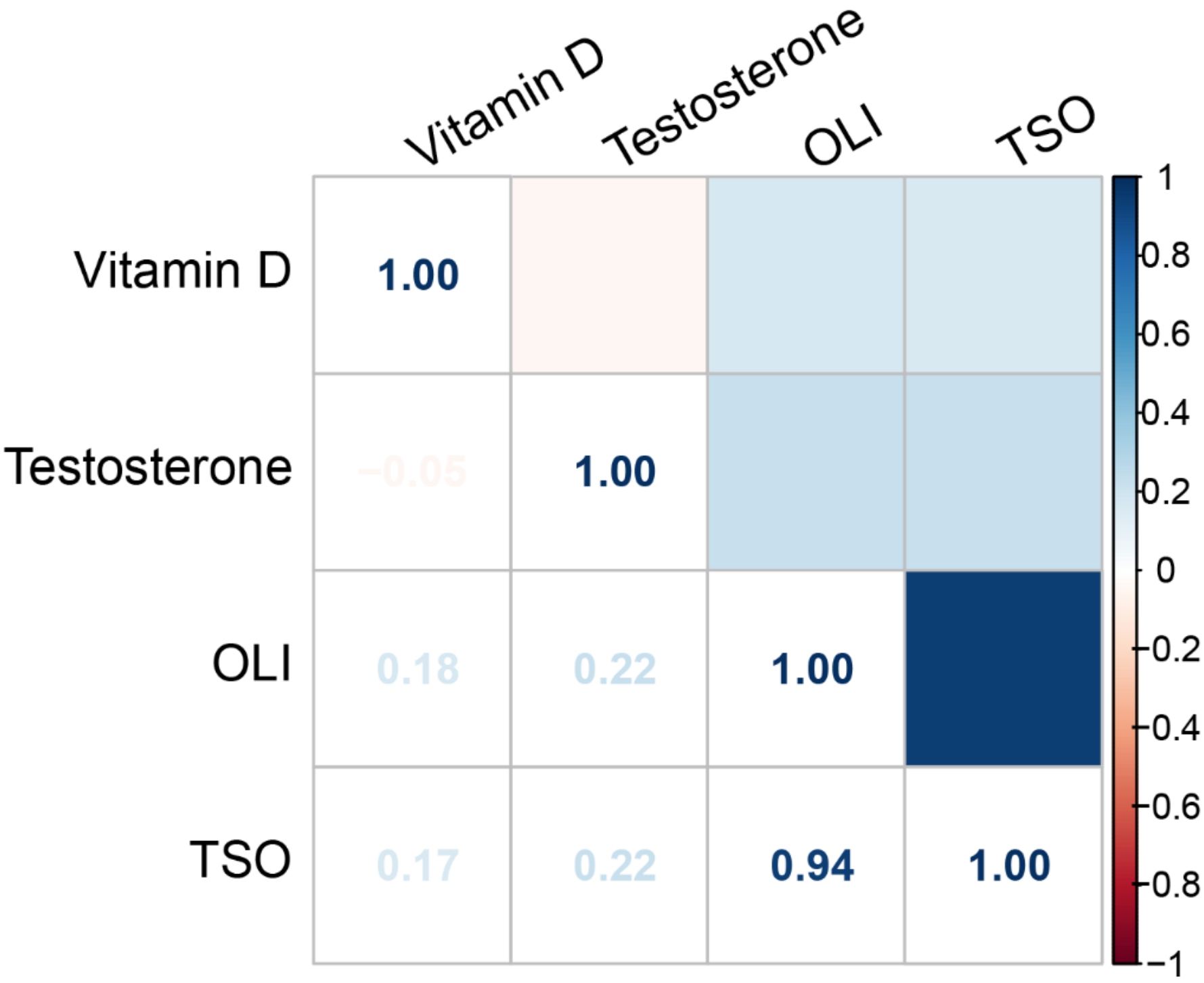

Supplementary Figure 2. The mixed mediation results of (A)TSO. (B)OLI. (threshold >500lux)

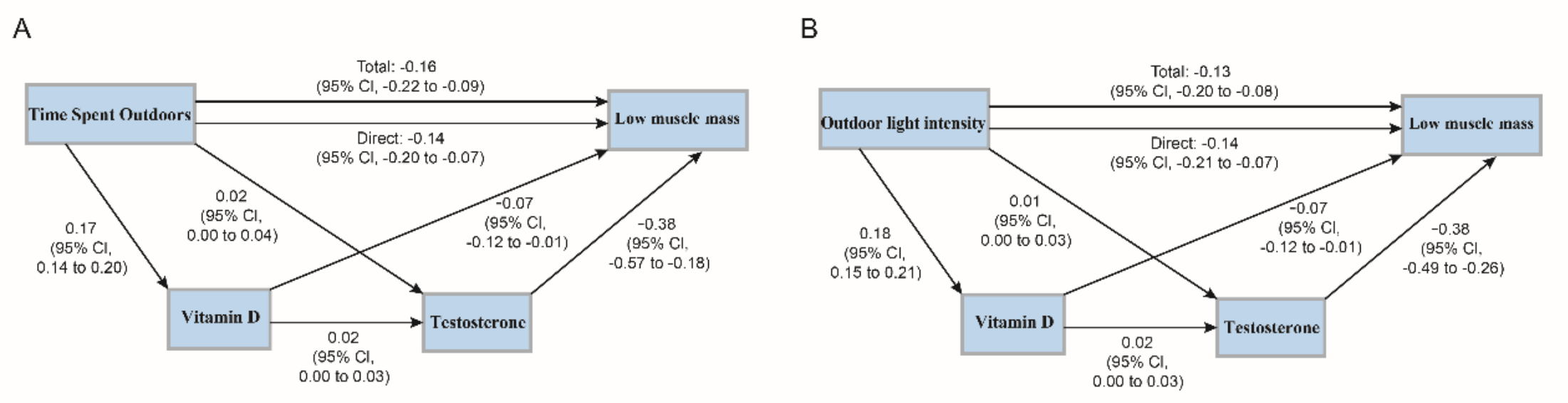

Supplement: Supplementary file 1 [file medi-105-e48257-s001.pdf]
