# Supplementary material for: Associations between outdoor light exposure and low muscle mass: An NHANES 2011 to 2014 analysis
Source: Medicine (Baltimore). 2026 Apr 10;105(15):e48257. doi: 10.1097/MD.0000000000048257 (PMC13593248; doi:10.1097/MD.0000000000048257)
Supplement: Supplementary file 2 [file medi-105-e48257-s002.pdf]

**Supplementary Table 1. The chain mediation path of "vitamin D → testosterone"**

Path Coefficient

| Relation | Label | β (95% CI) | Std. Error | z | pvalue |
|---|---|---|---|---|---|
| TSO → VITAMIN D | a1 | 0.17(0.14, 0.21) | 0.02 | 10.72 | 0.000 |
| VITAMIN D → TESTOSTERONE | a2 | 0.03(0.01, 0.04) | 0.01 | 3.28 | 0.001 |
| TESTOSTERONE → LMM | b | -0.39(-0.55, -0.23) | 0.08 | -4.74 | 0.000 |
| TSO → LMM | ADE | -0.13(-0.21, -0.06) | 0.04 | -3.45 | 0.001 |
| OLI → VITAMIN D | a1 | 0.18(0.15, 0.22) | 0.02 | 11.90 | 0.000 |
| VITAMIN D → TESTOSTERONE | a2 | 0.03(0.01, 0.04) | 0.01 | 3.21 | 0.001 |
| TESTOSTERONE → LMM | b | -0.39(-0.5, -0.27) | 0.06 | -6.78 | 0.000 |
| OLI → LMM | ADE | -0.15(-0.22, -0.09) | 0.03 | -4.42 | 0.000 |

Effect Decomposition -TSO

| EffectType | Label | Estimate | Std. Error | z | 95% CI | *P* value |
|---|---|---|---|---|---|---|
| Total ACME | total indirect | 0.00 | 0.00 | -2.44 | (0, 0) | 0.015 |
| Total Effects | total effect | -0.14 | 0.04 | -3.49 | (-0.21, -0.06) | 0.000 |
| Proportion of Mediation | prop mediated | 0.01 | 0.01 | 1.99 | (0, 0.03) | 0.047 |

Effect Decomposition -OLI

| EffectType | Label | Estimate | Std. Error | z | 95% CI | *P* value |
|---|---|---|---|---|---|---|
| Total ACME | total indirect | 0.00 | 0.00 | -2.64 | (0, 0) | 0.008 |
| Total Effects | total effect | -0.16 | 0.03 | -4.48 | (-0.22, -0.09) | 0.000 |
| Proportion of Mediation | prop mediated | 0.01 | 0.01 | 2.22 | (0, 0.02) | 0.026 |

Adjust for: sex, age, race, educational level, marital status, cancer, arthritis, daily MIMS, history of diabetes and smoking.

**Supplementary Table 2. Survey-weighted association of TSO and OLI with the odds of LMM (threshold >500lux).**

| | Crude model [a] | | Model I [b] | | Model II [c] | |
|---|---|---|---|---|---|---|
| | OR (95% CI) | *P* value | OR (95% CI) | *P* value | OR (95% CI) | *P* value |
| TSO | 0.78 (0.66, 0.92) | 0.004 | 0.71 (0.59, 0.84) | <0.001 | 0.75 (0.63, 0.90) | 0.004 |
| Q1 | Ref | | Ref | | Ref | |
| Q2 | 0.59 (0.42, 0.83) | 0.003 | 0.61 (0.42, 0.89) | 0.014 | 0.64 (0.43, 0.94) | 0.026 |
| Q3 | 0.73 (0.51, 1.05) | 0.091 | 0.65 (0.43, 0.99) | 0.044 | 0.73 (0.46, 1.14) | 0.150 |
| Q4 | 0.45 (0.29, 0.69) | <0.001 | 0.36 (0.22, 0.57) | <0.001 | 0.43 (0.26, 0.72) | 0.003 |
| OLI | 0.80 (0.67, 0.94) | 0.010 | 0.73 (0.60, 0.89) | 0.003 | 0.78 (0.64, 0.94) | 0.011 |
| Q1 | Ref | | Ref | | Ref | |
| Q2 | 0.63 (0.42, 0.95) | 0.027 | 0.59 (0.39, 0.90) | 0.017 | 0.62 (0.40, 0.97) | 0.040 |
| Q3 | 0.74 (0.50, 1.09) | 0.130 | 0.64 (0.40, 1.01) | 0.056 | 0.71 (0.44, 1.16) | 0.200 |
| Q4 | 0.42 (0.27, 0.66) | <0.001 | 0.32 (0.20, 0.53) | <0.001 | 0.39 (0.23, 0.65) | <0.001 |
